# Infection Probability Index: Implementation of an Automated Chronic Wound Infection Marker

**DOI:** 10.3390/jcm11010169

**Published:** 2021-12-29

**Authors:** Franziska Schollemann, Janosch Kunczik, Henriette Dohmeier, Carina Barbosa Pereira, Andreas Follmann, Michael Czaplik

**Affiliations:** Department of Anesthesiology, Faculty of Medicine, Rheinisch-Westfälische Technische Hochschule Aachen University, 52062 Aachen, Germany; jkunczik@ukaachen.de (J.K.); henriette.dohmeier@rwth-aachen.de (H.D.); cbarbosapere@ukaachen.de (C.B.P.); afollmann@ukaachen.de (A.F.); mczaplik@ukaachen.de (M.C.)

**Keywords:** chronic wounds, monitoring, thermal imaging, camera calibration, segmentation, region growing, image processing, infection probability index, IPI

## Abstract

The number of people suffering from chronic wounds is increasing due to demographic changes and the global epidemics of obesity and diabetes. Innovative imaging techniques within the field of chronic wound diagnostics are required to improve wound care by predicting and detecting wound infections to accelerate the application of treatments. For this reason, the infection probability index (IPI) is introduced as a novel infection marker based on thermal wound imaging. To improve usability, the IPI was implemented to automate scoring. Visual and thermal image pairs of 60 wounds were acquired to test the implemented algorithms on clinical data. The proposed process consists of (1) determining various parameters of the IPI based on medical hypotheses, (2) acquiring data, (3) extracting camera distortions using camera calibration, and (4) preprocessing and (5) automating segmentation of the wound to calculate (6) the IPI. Wound segmentation is reviewed by user input, whereas the segmented area can be refined manually. Furthermore, in addition to proof of concept, IPIs’ correlation with C-reactive protein (CRP) levels as a clinical infection marker was evaluated. Based on average CRP levels, the patients were clustered into two groups, on the basis of the separation value of an averaged CRP level of 100. We calculated the IPIs of the 60 wound images based on automated wound segmentation. Average runtime was less than a minute. In the group with lower average CRP, a correlation between IPI and CRP was evident.

## 1. Introduction

Due to both demographic changes and the epidemic of obesity and diabetes, the number of chronic wounds is increasing [1,2]. More than 1% of the German population suffers from chronic wounds, and the numbers are continually rising [3]. Elderly people are especially affected—8% of all residents in German nursing homes have chronic wounds [4]. In general, 1–2% of the population in industrialized countries is said to suffer from chronic wounds [5]. The affected person’s quality of life deteriorates due to pain, restrictions in the range of motion, and concerns about possible infections, among others [6]. The majority of chronic wounds show a rise of presence of bacterial biofilms which indicate infections [7]. Infections can lead to tissue destruction, delayed wound healing and other severe complications such as sepsis [7,8]. Septic patients often require additional surgical procedures, which can lead to further complications [9]. Besides human suffering, there is also an economical interest in improving wound management, as 1 to 3% of the healthcare costs are produced by chronic wounds [2]. Wound infections in particular include the highest costs among surgical complications [10].

Infection is characterized by heat (calor), redness (rubor), swelling (tumor), pain (dolor) and loss of function (functio laesa) [11,12]. A wound makes the body vulnerable to microbes, such as germs, viruses and fungi [13]. Due to a dysfunction of the repair process [14], a wound can become chronic, enabling microbes to colonize and propagate within the wound bed [13]. However, even if all wounds are contaminated, not every wound evolves into an infection [12]. Treatments, including clinical dressings, are used to improve regeneration, prevent infection and maintain the moisture of a wound [15].

In clinical practice, the wound monitoring is done using optical observation (tumor, rubor), physician-patient consultation (dolor) and measurement of temperature (calor). The latter temperature determination is for example done by imposition of hands by the practitioner [16]. The sensing of local temperature changes can therefore be used for diagnosis, for example in diabetic foot ulcers [17]. Additionally, wound scores can be calculated manually to indicate a possible infection [18].

In addition, semi-quantitative systems are used to support clinical wound management. One application is the use of technical devices or software for wound documentation.

On the part of the camera manufacturers there are, for example, examination cameras, which, however, do not integrate image analysis. Examples of this are the DE605 General Examination Camera (Firefly Global, Belmont, MA, US) and the General Examination Camera AMD-2500 (AMD Global Telemedicine, Chelmsford, MA, US) among others. Florczak et al. introduced a camera-based mobile device, which can be used to store images of the wound as well as further details of the wound and the patient [19]. A similar system is commercially published by the company XOTOTEC (XOTO Technology GmbH, Heiligenhaus, Germany). Wound documentation is also available in the form of a mobile app, for example the WundDoku App (Dr. Ausbüttel & Co. GmbH, Dortmund, Germany) or the WoundCareLog APP introduced by Dong et al. [20]. Another approach included a decision support algorithm to support the user in therapy decisions [21]. However, even though these approaches can be used to improve the clinical process, standardize the wound management and save time [22], they only serve as passive documentation and have no added value with regard to infections.

Other semi-quantitative systems address the contactless wound measurement. Planar, 2D images can form the basis to measure the wound’s width, height and area [23,24]. Moreover, there are multiple publications using contactless measurement to generate a 3D image of the wound to improve accuracy [25,26,27,28]. This measurement was for example integrated in a mobile application [29]. Wang et al. introduced wound measurement combined with a thermal camera but only to compare the temperature measured with the result of a contact-thermometer without any further analysis of the thermal data [30]. These wound measurement approaches can be used to extend the wound documentation; however, they do not give any insights regarding infection.

Therefore, further solutions are needed to detect wound infection to initiate treatments at an early stage. For this purpose, smart bandages including biosensors have been introduced to monitor parameters such as temperature, due to its coherence with infection [15,31,32,33]. Hsu et al. used visual images to classify wounds into categories including infection [34], but without using thermal data. Barone et al. introduced a system containing thermal, 3D, and visual data to measure and classify wounds [35]. However, there was no detection or prediction of infection.

Nevertheless, the use of thermal analysis in connection with wound monitoring is well justified. Based on calor (heat), local increase in temperature difference is likely coherent with inflammation or—in the presence of an injury—might be a sign for an infection [36,37]. Within research, due to the coherence of infection and local temperature changes in venous leg ulcer patients, absolute temperature values of the wound were compared with the clinical wound bed score [36]. Even though these wounds are different from the ones used here, Dini et al. show a correlation between temperature and infection. It was also shown that the difference between the wound temperature’s maximum and the reference skin temperature was higher for infected wounds compared to those that were healing [37].

Blood analysis is used to measure inflammation markers such as C-reactive protein (CRP) and white blood cells [38]. CRP involves an acute inflammation protein, the level of which can increase due to an infection [39]. The CRP value is measured with the aid of a blood sample, for example, using a heterogeneous immunoassay [38]. In analyzing the CRP level, the blood chemistry parameter is used to evaluate a treatment’s success regarding wound infections, among other treatment evaluations [39]. As a matter of fact, CRP levels depend on the extent of systemic response and thus also on the severity of wound infection. However, the CRP value is a global inflammatory marker [40], which in general is elevated for ICU patients [41].

Although some links are drawn between the analysis of thermal data and infections, this knowledge is not yet used in practice. Existing routines are based either on manual scoring or on technology that is used for wound documentation. Even though the measurement of a wound is helpful for documentation, it cannot detect nor predict an infection. Hence, there is a great demand for solutions that automatically analyze the current status of wounds and predict whether an acute or chronic wound is healing physiologically or if there is a tendency towards infection [42].

This paper presents a new approach to detect and predict infections in acute and chronic wounds and monitor wound healing using thermal data as a proof of concept. Three scientific goals are pursued: (1) Contrary to other approaches, a novel infection probability index (IPI) is introduced, which, in addition to the temperature difference, includes other thermal wound characteristics. (2) To improve clinical relevance and to simplify the application, the IPI score is automated using image processing. The introduced algorithms include preprocessing including body segmentation, automated wound segmentation, and calculation of the IPI. (3) For an initial evaluation, the IPI is compared with an existing clinical parameter to demonstrate the potential of its use in clinical practice.

## 2. Materials and Methods

### 2.1. Infection Probability Index

The IPI was developed by an interdisciplinary team, consisting of five physicians and three biomedical engineers. Hypotheses were formulated by the physicians, based on (1) medical foundation and the state of knowledge regarding the temperature characteristics of wounds, as well as (2) the manual analysis of thermal images of different wound types. Here, the focus was on analyzing a broad spectrum of wounds that tend to run a chronic course in order to develop a score that is as generally valid as possible for chronic wounds. The wound types considered are described in more detail in the following paragraph. Regarding nomenclature, the following definitions were set:

- The wound base forms the center of the wound.
- The anatomical wound margin is visible to the naked eye, surrounding the wound base.
- The thermal wound margin comprising the wound area within the thermal image.

Within an iterative process, the determined hypotheses were discussed, refined or partially discarded. Based on the manual evaluations of the images, the various hypotheses were weighted differently for the score with regard to their relation to potential infection. The interdisciplinary team was used to include the engineers within the development of the IPI to improve the latter implemented automation of the IPI score.

#### Wound Types

Wound types including chronic wounds in form of pressure injuries, as well as acute wounds after laparotomy, or non-healing stomata were considered within the development of the IPI. Pressure injuries occur also but not exclusively due to pressure. Additional factors of influence are malnutrition or inadequate supply of nutrients, for example, related to weight loss due to illness [43]. Additionally, age and mobility impairments can contribute to a pressure injury [44,45]. However, there are some predictable pressure points that can trigger a pressure injury (occiput, scapula, elbows, sacrum, ischium and heel), which is why it is recommended to change ICU patients’ positions every 2 h [44]. Even though the number of observational studies has increased in recent years, only a marginal number have dealt with the prevention and treatment of pressure injuries [46]. Regarding the international guideline for pressure ulcer, the stage 1 pressure ulcer wound bed is defined as to be warmer or cooler compared to the adjacent areas, among others [47]. A problem with pressure injuries is that they tend to become chronic and, even if they heal, to recrudesce [48]. In contrast to pressure injuries, acute wounds after laparotomy and stomata arise from surgical interventions. The aim of a laparotomy is to open the abdomen by means of electric scalpel or surgical scissors and knives [49], for example, for hemostasis and controlling peritoneal spillage after an intestinal lesion [50]. Stomata can be triggered by trauma, as a majority of injuries based on external forceful impact are abdominal trauma [51]. Here, laparotomy is a standard surgical procedure, whereas the use of a more minimally invasive approach is becoming common [49]. If the complaint returns, another laparotomy (relaparotomy) can also be necessary [49]. A possible consequence of laparotomy is a surgical site infection (SSI), which is even more likely if a stoma was surgically created [52]. The aim of a stoma is to surgically open a segment of the intestine through the abdominal wall to create an artificial anus for excretion [53]. Since infection can also occur in this case, possibly leading to ischemia or necrosis, detection of pathological wound healing is also crucial [54].

### 2.2. Automation of the Infection Probability Index

To enable the automated calculation of the IPI, several steps were required for preparation using algorithms implemented in Python (Python Software Foundation, Wilmington, DE, USA). These are presented within the following chapter. For the segmentation of the wound, a visual image was also acquired in addition to the thermal image. Therefore, an image pair comprised one thermal and one visual image, which were acquired using a multimodal camera. The visual or RGB image can be divided into three channels: red (R), green (G) and blue (B), which were used for parts of the research as individual images. Regarding implementation of the algorithms, a test data set consisting of 15 image pairs was formed. After the data acquisition, camera distortions of both thermal and visual images were eliminated using camera calibration [55,56]. Within the preprocessing, segmentations of the human from the background for both image modalities were determined. The anatomical wound margin was segmented automatically within the visual segmentation and confirmed by user input. Then, as the result, the IPI was calculated automatically and compared to the CRP level within the statistical analysis.

#### 2.2.1. Data Acquisition

The data were acquired in a clinical study conducted in the University Hospital Aachen. For this, a FLIR E95 thermal camera (FLIR Systems Inc., Wilsonville, OR, USA) with a resolution of 464 × 348 px and a thermal sensitivity better than 0.04 ${}^{\circ }$C at 30 ${}^{\circ }$C was used. The images were shot from a distance of 30 cm to the patient, while it was made sure that no identifiable body parts (for example the head) were in the picture.

#### 2.2.2. Camera Calibration

Within this multimodal approach, both visual and thermal images were used to calculate the IPI. Therefore, camera calibration was an essential task to perform before the actual image processing to eliminate camera-specific distortions. Regarding the visual images, checkerboard patterns could be used to calculate the camera parameters, based on the pattern from MATLAB (The MathWorks, Inc., Natick, MA, US). Based on the dependence of the emissivity factor on the brightness of the surface, the black and white squares of the checkerboard pattern, warmed by a heat lamp, could be distinguished within the thermal image [55]. Therefore, the checkerboard pattern could simultaneously be used for thermal camera calibration. Both thermal and visual images were analyzed separately by the MATLAB-integrated camera calibrator app. The generated thermal and visual camera parameters were imported into Python and used to adjust both thermal and visual images using the OpenCV (Intel, Santa Clara, CA, US) undistort function.

#### 2.2.3. Preprocessing

The aim of the preprocessing (Algorithm 1) was to segment the skin area within both image types using a mask. A mask was defined as a binary image in which the value “1” included the pixels to be considered and “0” the background, and thus the irrelevant part of the image. A mask can be used within this process, for example, to calculate the mean value of an area by multiplying the mask and the original image. Whereas the process for calculating the skin mask, the segmentation of the human within the image, was the same for both modalities beginning with an initial segmentation of the skin area, the latter was separately calculated for both modalities. Regarding the thermal image, a k-means approach was used to divide the image into clusters [57]. The mean of the clusters’ centers was used to threshold the image to segment the skin, as the skin’s temperature is higher than the temperature of the background. Based on experimental results, a lower threshold of 30 ${}^{\circ }$C for human skin was ascertained. Hence, if the threshold calculated by the k-means approach was higher than 30 ${}^{\circ }$C (for example, due to a very hot wound area or a significant amount of skin and wound within an image), the threshold was corrected to 30 ${}^{\circ }$C. Expressed as a formula using the threshold of the k-means approach *T*, the initial segmentation of the thermal image can be described as

(1) $${\mathrm{mask}}_{\mathrm{thermal}}(x,y)=\left\{\begin{array}{c} 1\;\mathrm{if}\;\mathrm{Temperature}\left(x,y)\geq \min[T,30\right]\\ 0\;\mathrm{if}\;\mathrm{Temperature}\left(x,y)<\min[T,30\right] \end{array}\right.$$

Prior to the development of the algorithm, a manual analysis of the visual images was performed by the biomedical engineers to derive differences within the image regions as a basis for segmentation. During this analysis of the various image regions (classified as “wound area”, “skin area”, “human (e.g., clothes) area”, “medical components” and “background”), the hypothesis was that the R (red-channel) value would represent a maximum of all three values within the skin area. Analogous to Equation (1), the initial skin segmentation within the visual image can be described as

(2) $${\mathrm{mask}}_{\mathrm{visual}}(x,y)=\left\{\begin{array}{c} 1\;\mathrm{if}\;R(x,y)=\max[R(x,y),G(x,y),B(x,y)]\\ 0\;\mathrm{if}\;R(x,y)\neq \max[R(x,y),G(x,y),B(x,y)] \end{array}\right.$$

The biggest area was selected within both the thermal and the visual initial skin mask. To improve the initial skin mask, a combination of region growing and edge detection was integrated. The combination of these approaches was used in different ways [58,59,60,61,62,63]. The benefit of integrating both the edge and local information was to refine the result at the border of the mask. Therefore, the general aim was to use the previously calculated skin mask as a basis to calculate a region-growing algorithm to make the mask grow against the edges.

Therefore, the Canny edge detector by John F. Canny was used to find edges in images; it was applied to the grey images of the original visual and thermal images to reduce complexity [59]. Within region-growing algorithms, seed pixels or areas are used to check whether the seeds’ neighbor pixels fulfill defined conditions [60]. As a seed point, a core (10% of its original size) of the initial skin mask was calculated. The region-growth condition was that the pixel to be considered was part of the initial skin mask and was not labeled as an edge.

**Figure 1:**
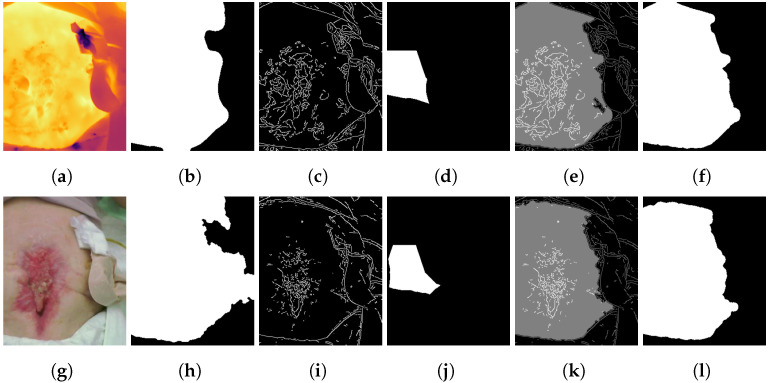
Calculation of the skin mask. (**a**,**g**) Original thermal/visual images, (**b**,**h**) initial skin mask based on Equation (1)/(2), (**c**,**i**) edges based on canny algorithm, (**d**,**j**) core of initial masks, (**e**,**k**) region growing based on core of initial mask and edges as condition and (**f**,**l**) final skin mask.

The biggest area was considered the final skin mask for both images. Within the last step of preprocessing, the mask was “cleaned” using morphological operations, particularly dilation and erosion. While using dilation for to expand a mask could lead to filing holes or connecting various regions within this mask, erosion is used to shrink an mask (as performed for calculating the core of the initial skin mask) [64]. Kernels were necessary to the morphological operation, for example, a 3×3 matrix. A combination of erosion and then dilation using the same kernel size could lead to a smoothing of the border of a mask and to deleting singular pixel-sized areas. Furthermore, possible holes in the mask were filled. The work flow of the preprocessing algorithm is shown at Algorithm 1, whereas the steps described are illustrated by two example image sets in Figure 1.
**Algorithm 1:** Preprocessing.1:**procedure**Skin mask segmentation2:    **for** input=[thermal,visual] **do**3:        **if** input=thermal **then**4:           image=thermal5:           T=k-means(input)6:           **if** I(x,y)≥min[T,30] **then**7:               mask(x,y)=18:           **else**9:                mask(x,y)=010:           **end if**11:        **else**12:           image=visual13:           **if** R(x,y)=max[R(x,y),G(x,y),B(x,y)] **then**14:               mask(x,y)=115:           **else**16:               mask(x,y)=017:           **end if**18:        **end if**19:        edges=canny(image)20:        core=erosion(mask,10%)21:        mask=regionGrowing(seeds=core,condition=[mask=1,edges=0])22:        mask=segmentBiggestArea(mask)23:    **end for**24:**end procedure**

#### 2.2.4. Wound Segmentation

In this section, the steps within the wound segmentation procedure (Algorithm 2) are explained. The visual image was used to perform the wound segmentation. In this modality, the wound margin had better contrast compared to a thermal image. The aim of this algorithm is—in contrast to the preprocessing before—to segment the wound area within the previous calculated human mask.
**Algorithm 2:** Wound segmentation.1:edges=canny(G)2:IDs=labeling(edges)3:meanTemperature=average(mean(edges,thermal))4:**for** iter = 1:length(IDs) **do**5:    ID=IDs(iter)6:    **if** mean(edges(ID),thermal)>meanTemp **then**7:        edges(ID)=08:    **end if**9:**end for**10:woundMask=regionGrowing(seeds=edges,condition=[valuedarkerthanseed])11:woundIDs=labeling(woundMask)12:selectedIDs=userinput()13:**if**userinput=0**then**14:    woundmask=manualSegmentation()15:**else**16:    woundmask=woundmask[woundIDs==selectedIDs]17:**end if**

An inspection of the visual test images showed that the green channel yielded the highest contrast between normal skin and the wound area. Therefore, a Canny edge detection was computed using the G channel image. An opening morphological transformation was then used to clean the resulting borders and delete speckled pixels. All detected edges were then labeled.

The next step was to identify edges of the actual wound margin. Local necrosis, crusts, or fluids inside a wound area also create edges. To differentiate, the mean temperature along all edges was evaluated because the wound temperature at the margin is lower than inside the wound itself. Only edges with a mean temperature below the total averaged mean value were considered relevant.

**Figure 2:**
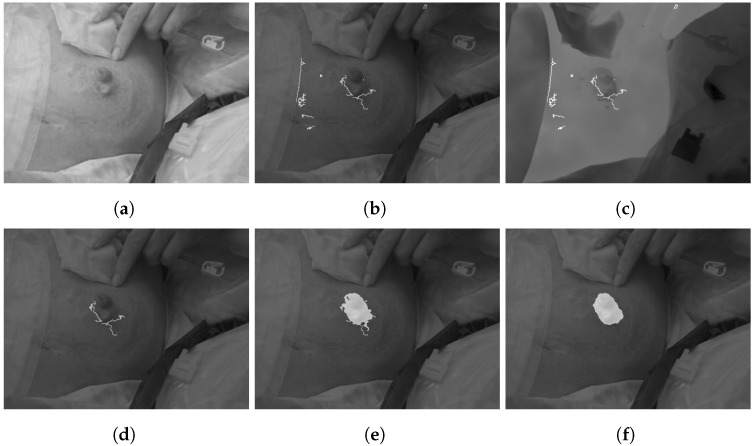
Segmentation of the wound margin. (**a**) Original visual image, (**b**) edges based on canny algorithm, (**c**) overlay of thermal image and edges, (**d**) edges after temperature thresholding, (**e**) region growing based edges as seeds and (**f**) final wound mask.

Because the aim was to segment the whole wound area, a region-growing algorithm was implemented, using the edges as individual seed points. Therefore, based on the labeled edge mask, region growing was performed for each region of the edge mask. The condition for growing was determined as an equal or darker value related to the seed pixels, and as compared to normal skin, the wound area appeared darker within the visual image. The described steps of wound segmentation are visualized in Figure 2 using an example image.

The wound segmentation was complemented with a final manual review step that enables the clinical staff to select the relevant segmented wound margin. This step was necessary because there might be several wounds within one image or other anatomical shapes that could also be segmented as a wound (for example, a nipple). Simultaneously, the possibility was implemented of segmenting the wound manually through user input if the segmentation was not sufficient.

On this basis, the implementation of the IPI was carried out, which is described in the following chapter.

### 2.3. Comparison of the Infection Probability Index to a Clinical Parameter

Regarding an initial evaluation of the potential of the introduced infection probability index, the automatically calculated score was compared with the measured CRP-level. Even though the exact value of the IPI is relevant in later practice for a warning in case of an increase, the first step was to compare the correlation between the assessment of the IPI (no infection/infection) and the laboratory value in the form of the averaged CRP level. For this reason, the IPI was divided into two clusters: Probably no infection (1–4 points) and potential infection (5–9 points). Similarly, the patients were clustered into two categories based on the averaged CRP level. This had a medical background, as the CRP level with a larger open wound can basically be higher in general than that of a smaller injury. Therefore, the patients were analyzed in terms of their averaged CRP level in two clusters with the threshold CRP value 100.

#### Statistical Analysis

IBM SPSS Statistics 24 was used for statistical analysis. The significance level chosen was *p* = 0.05. The analysis of data correlating the clustered CRP and IPI was done using Spearman’s Rho correlation coefficient.

## 3. Results

The core of this paper was the elaboration of the IPI based on medical hypotheses informed by thermal images. Therefore, various characteristics of a potentially infected wound were extracted and are described in Section 3.1. The resulting IPI was automated in the next step. In addition, the IPI values are compared with clinical data.

### 3.1. Infection Probability Index

A number of thermal and visual images were analyzed manually with experienced clinicians to elicit robust thermal parameters indicating infections. The resulting four parameters are described in the following subchapters:

- Cold spots (punctual within wound base);
- Temperature difference (wound to intact skin);
- Temperature distribution (within the wound base);
- Thermal wound margin.

These parameters were weighted, as illustrated in Table 1. In total, the IPI has a point scale from 0 (no potential infection) to 9 (potential infection) points.

#### 3.1.1. Cold Spots

As one potential marker for infection, cold spots within the wound region were determined. These were defined as small, selective areas of significantly colder temperature compared to the mean wound temperature, which were often found within potentially infected wounds. The mean value of these areas was often more than 2 ${}^{\circ }$C colder than the wound’s mean temperature. Moreover, a higher number of them corresponded to a higher possibility of wound infection. Due to a high relation between cold spots and possible infection, the medical practitioners doubled the weight of this parameter.

#### 3.1.2. Temperature Difference

Based on the state of the knowledge regarding temperature difference as well as the manual analysis, the temperature difference between the wound and unimpaired skin was considered a marker for potential infection.

#### 3.1.3. Temperature Distribution

Temperature distribution within the wound base also seemed to be a marker for possible infection. Regarding a physiological healing wound, the temperatures within the wound base were very homogenous, according to the hypothesis. In contrast, the temperature distribution was inhomogeneous or concentrated within a possibly infected wound.

#### 3.1.4. Wound Margin

Within the analysis of thermal wound images, a characteristic of possible infection regarding the thermal wound margin was determined. Compared to a physiological healing wound, where the thermal wound margin surrounded the wound equidistantly, the thermal wound margin would cross the anatomical wound margin in a pathological healing wound. Hence, the parameter of a discontinuing thermal wound margin was added.

### 3.2. Automation of the Infection Probability Index

The implementations of the previously described IPI parameters are described for automated evaluation within this section. This process was used to score 60 acquired longitudinal image pairs of 7 patients within 9 months (April 2019–January 2020). The data set for this study consisted of 2 pressure injuries, 3 wounds after laparotomy and 6 stomas, in total 11 wounds. The amount of images per wound was between 2 and 17 (IQR[3;4]). Moreover, the CRP-level was measured and averaged for each patient (see Table 2).

The implemented algorithms were used to analyze the images. The averaged runtime of the process starting before loading the images was 44.4 seconds (IRQ[36.73;53;24]). Wounds of 57% of the images were segmented automatically; 40% had to be refined manually, and for 3%, the wound was not found correctly and therefore was segmented manually.

Within this multimodal approach, there was a difference between the visual and the thermal images based on the physical offset of the two cameras. Even though the skin mask was calculated for both image modalities, the registration of these two masks failed to improve the spacing in image detail. However, due to the small distance between camera and patient, this spacing was averaged over the test dataset 10 pixel (2.24% of image width, 2.99% of image height).

#### 3.2.1. Cold Spots

Regarding the implementation, the mean temperature value of the segmented wound base area was calculated. By subtracting the mean temperature value of the wound base, the areas below the temperature threshold (Table 1) could be determined, establishing the potential cold spots. Labeling these areas may help verify if the size of each potential cold spot was within the limit values. The lower size threshold was defined to prevent singular-pixel error segmentation and was set to 10 pixels. Due to the medical definition of cold spots as punctual areas, there was also an upper threshold set to 100 pixels. The number of resulting cold spots was then calculated, and the cold spot IPI value estimated based on a case distinction (Table 1).

#### 3.2.2. Temperature Difference

The maximum temperature of the wound base was estimated. Moreover, to determine the temperature of unimpaired skin, the thermal wound margin was computed. Then, the anatomical wound margin based on the border of the wound mask was divided into super pixels. These super pixels gathered adjacent pixels with a similar temperature value, and their mean value was calculated to reduce complexity. For each super pixel, the thermal wound margin was calculated using the super pixel as a seed point for a region-growing algorithm defining the border of a 1.5 ${}^{\circ }$C drop in temperature. The resulting thermal wound margin is then dilated to establish the area of the impaired wound area and multiplied with the skin mask. Regarding the IPI value, the temperature difference between the calculated maximum temperature of the wound base compared to the impaired skin area was computed, and the IPI value weighted (Table 1).

#### 3.2.3. Temperature Distribution

The wound base and mean temperature values were determined to check homogeneity. If the difference of an individual pixel’s temperature from the mean value differed from the set value, the pixel was considered inhomogeneous. Therefore, the number of inhomogeneous pixels was calculated, and depending on the majority, the wound base was defined as inhomogeneous or homogeneous. Regarding the concentrated temperature distribution, the wound base was divided into super pixels to reduce computing time and complexity. Using an adapted region-growing algorithm, the super pixels were united with adjacent super pixels if the difference of their mean values were beneath 1.5 ${}^{\circ }$C. For the newly created area, the mean value was calculated based on the previous mean temperatures and sizes of the two areas. The size of the resulting areas was evaluated and weighted based on case discrimination (Table 1).

#### 3.2.4. Wound Margin

Similar to the temperature difference, the thermal wound margin was calculated using the region-growing algorithm based on super pixels of the anatomical wound margin. Afterwards, a possible crossing of the resulting thermal wound margin and anatomical wound margin was determined by calculating an adapted region-growing algorithm based on the surrounding seed pixels growing inside the wound, if the latter was not regularly “guarded” by the thermal wound mask.

### 3.3. Comparison of the Infection Probability Index to a Clinical Parameter

**Figure 3:**
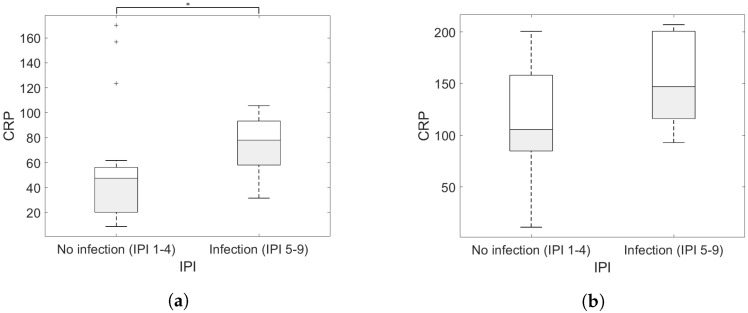
Boxplot of the IPI and the CRP level for both CRP clusters. (**a**) Cluster 1: averaged CRP level of the patient of less than 100. (**b**) Cluster 2: averaged CRP level of the patient of higher than 100.

Regarding the first cluster (averaged CRP of the patient < 100), the correlation was significant with a correlation coefficient of 0.331 with a *p* value = 0.037, see Figure 3. The analysis test of the second cluster (averaged CRP of the patient > 100) resulted in a correlation coefficient of 0.221 with a *p* value > 0.05, hence no significant correlation.

## 4. Discussion

In this research, the infection probability index to detect wound infection was elaborated, automated using image processing techniques, and finally compared to a clinical parameter.

During the development of the medical hypothesis, the thermal analysis was already used on different wound types to broaden possible applications. Therefore, it was necessary to define possible thermal characteristics relating to infection that could be used for chronic wounds of different origins. Regarding the state of knowledge, the analysis of absolute wound’s temperature or temperature difference was also used on different chronic wound types as for example leg ulcer [36], abdominal surgical wounds [65], as well as pressure ulcer and amputation wounds [37]. In this research, images of three different types of wounds that tend to run a chronic course were analyzed: pressure injuries, stoma and wounds after laparotomy. These wound types are differentiated by origin, shape and size of the wound. Pressure injuries are generally classified as chronic. Laparotomy wounds are initially acute but can also become chronic if healing is disturbed, for example, by infection or other disorders. An artificially created stoma, on the other hand, is often not initially considered a wound—but can also become chronic if physiological healing does not occur. At the same time, despite their differences, they showed similar thermal characteristics, which were summarized within the IPI parameters. For this reason, parameters have been developed which can be applied to wounds of different sizes to detect infection because infection is independent of wound size. In addition, there are already some suitable systems for documenting the geometric dimensions of the wound [23,24,25,26,27,28,29]. However, since this information does not provide information about a possible infection, it is important to aim for an additional broadest possible infection detection. In previous work, the link between thermal wound characteristics and infection was shown [36,37]. Since the IPI presented here includes different thermal conditions, it goes one step further than, for example, individual temperature measurements or difference analysis [36,37,65]. Moreover, compared to visual images, thermal imaging includes the advantage of an information gain (calor), since visual cameras can only capture what can also be visually analyzed on site. As a matter of consequence, the approach presented here can be distinguished from classification of infection within visual images [34]. Similarly, Barone et al. integrated thermal imaging into the multimodal system; however, there was no infection detection [35]. As a result, medical hypotheses based on thermal images were compiled, and the four IPI categories were derived: (a) temperature difference, (b) cold spots, (c) temperature distribution and (d) wound margin. This resulted in a novel thermal score that does not exist in standard clinical practice to this day. Regarding temperature difference, Dini et al. compared, in contrast to us, the absolute temperature value with the bed score [36]. However, the use of relative temperature differences offers the advantage of being able to intercept general variations in the images from the thermal camera and thus increase accuracy. Under the hypothesis that the temperature of the intact skin is independent of the wound, since it is not in the vicinity of the wound, it is possible to relate the correlation to infection also to the temperature difference. The medical hypothesis regarding the temperature difference was that for normal wound healing, the temperature difference is between 1 and 2 ${}^{\circ }$C. This is supported by Chanmugam et al., where with a comparable study setting, the difference was 1.2–1.3 ${}^{\circ }$C within the control, non-infection group [37]. Accordingly, the difference of wound temperature to a reference temperature—in this case the intact abdominal temperature—of the non-infected group was measured as 1.12 ${}^{\circ }$C, 1.09 ${}^{\circ }$C and 1.56 ${}^{\circ }$C on the second, seventh and fifteenth days postsurgery, respectively [65]. In our case distinction, a temperature difference above 2 ${}^{\circ }$C is a possible sign of infection. This threshold formation also includes the result of Chanmugam et al., where the temperature difference was even 4–5 ${}^{\circ }$C with clinically diagnosed wound infection [37]. Regarding the difference of wound temperature to the abdominal temperature of the infected group, the measurements were 1.73 ${}^{\circ }$C, 1.92 ${}^{\circ }$C and 1.95 ${}^{\circ }$C on the second, seventh and fifteenth days postsurgery, respectively [65], which is smaller than the limit we set. However, influences such as necrosis which lower the mean wound temperature could not be taken into account by using only one threshold. This also applies, for example, to the result of Chanmugam et al., according to which antibiotic-treated wounds had a temperature difference that was lower than the control group [37]. For this reason, we introduced a further limit: if the temperature difference is less than 1 ${}^{\circ }$C, this might illustrate a pathological wound healing, for example, necrosis. Due to the usage of thermal images compared to thermal measurement for example using a thermometer, it was possible to not only measure the temperature but also analyze and work out further thermal characteristics. These include both the wound base (cold spots and temperature distribution) as well as the peri-wound area (thermal wound margin). The cause of the cold spots could be accumulated fluids, for example, purulence, or exudate. They occurred within the establishment of medical hypotheses regardless of the size of the wound. The definition of a range, which the size of cold spots can assume, also contributes to the fact that similar characteristic cold spots can be found in the different wound types. Accordingly, the analysis of the temperature distribution within the wound base was possible for the different wounds. Barone et al. also used segmentation to classify regions of similar temperature within the thermal image [35]. However, there was no infection analysis or further conclusion regarding the resulted segmentation. The multimodal approach using both visual and thermal images allowed the analysis of the thermal characteristics of the wound base but also of the peri-wound area [35]. Fierheller and Sibbald calculated the temperature difference between the peri-wound temperature and the intact skin [16]. However, as shown for example in Figure 1a, the peri-wound area differed depending on the place of consideration, which is why a simple temperature difference calculation seemed to not capture all the information. In many physiological healing wounds, the thermal wound margin was equidistant, formed around the wound base. However, in potential infection images, the thermal wound margin was discontinuous and crossed the anatomical wound margin. This analysis of the holistic peri-wound area using the thermal wound margin compared to a single temperature measurement can also be reinforced by clinical practice, in which the physicians might touch multiple different areas close to the wound.

Since a manual analysis of the images would imply additional effort within possible future applications, the scoring was automated by implementing preprocessing, segmentation and scoring algorithms. As a result, a fully-automated analysis was introduced. The averaged runtime to preprocess, segment both the skin and the wound mask and calculate the IPI was less than a minute on a standard laptop. The runtime of the algorithm can be reasoned with the several working steps as well as the iterative calculation of, for example, the region growing algorithms. However, since the whole process is automated (except for the user input based on the wound segmentation) the proposed algorithms are practical due to no significant additional expenditure for the user. Thereby, the IPI provides a gain in information without significant additional effort. Even though otherwise mainly temperature differences were calculated, an absolute temperature value was used as a threshold: within the segmentation of the visual image, the threshold was set to 30, if this would otherwise be exceeded. The advantage over a relative threshold—for example, using a percentile calculation—in this particular case was that this also worked for images whose image section consists mainly of skin and wound. For the latter, a relative, percentile threshold would result in an incorrect classification, segmenting part of the human as background.

The wound segmentation based on the visual image was validated by user input, and therefore, the IPI of 60 image pairs was calculated. A large part of the wounds were segmented automatically, whereas the evaluation of the different IPI categories worked without problems. For potential correction purposes, a possibility to refine or replace the automated wound segmentation was integrated as a user input.

However, there was a connection between increasing IPI and CRP-value. Correlation within the first CRP cluster revealed a significant relationship between IPI (no infection/infection) classification and CRP levels. In contrast, the correlation in the second cluster was not significant. The aim here is not to prove the correlation between temperature and infection, which was already done in several publications and formed the basis for the use of temperature sensors. Moreover, an increased CRP level indicates a general inflammation without pointing towards a specific infection [39] nor a specific wound [40], if the patient for example has multiple wounds. Other infections can also increase the patient’s CRP level—e.g., infections such as urinary tract infection or upper respiratory tract infection, which have no connection to wound healing. For this reason, CRP is a very non-specific laboratory value, which is why the IPI needs to be evaluated in further studies.

With reference to the limitations of the paper, it is necessary to consider several aspects. The intention of this paper was the proof of concept of the automated IPI, wherefore the weights of the parameter have to be refined using a bigger dataset. Moreover, the various parameters of the IPI could be analyzed based on their individual correlations to infection.

First, the sample size of the patients is low, wherefore further studies are needed. The offset between the visual and thermal images could not be improved automatically. However, since no punctual measurement but the investigation of a whole area was undertaken, this spacing was considered tolerable. Based on the issue that the concise points differ in thermal and visual images, reference markers, for example, could be applied to the skin [66]. However, this would decrease the benefits of a contactless wound analysis due to a possible spread of infection [67] as well as result in higher time investment for the nursing staff. Wound segmentation worked automatically for most of the images. However, it was based on the hypothesis that the RGB-values of the wound area were darker than the skin area, which must be tested for a larger number of images of patients of different skin color.

Within the statistical evaluation, it is important to note that dependent and non-dependent variables were compared. Another problem is that CRP is a global value. For patients with multiple wounds, which were scored using the IPI individually, the CRP value was influenced by all wounds. Thus, considering a patient with multiple wounds, a high CRP value might be associated with a non-infected wound if one of the other wounds were conspicuous. Due to the focus of the paper and the limitation of the available data, the relationship between IPI and infection needs to be investigated in further studies. Additionally, the proposed analysis was performed on a standard laptop after the data acquisition. It would be much more practicable to integrate the proposed algorithms into a system to enable direct analysis.

As an outlook, an important next step would be to integrate the proposed algorithms into a mobile device. Thereby, the analysis of the images could be done directly at the patient’s bedside to improve usability. Furthermore, more modalities could be added, for example, a depth camera. Based on the distance measurement of a depth camera, the limitation of the spacing in image detail between the visual and thermal images could be solved. By including a depth camera, the measurement of visual wound characteristics (e.g., wound size, shape) over time could also be possible [23,24,25,26,27,28,29]. The software foundation for this mobile system could be the algorithms proposed within this research as a proof of concept. At the same time, the IPI could be supplemented with other potential parameters. For example, information based on the visual image as for the classification into the groups granulation, necrosis, fibrin and pus could be integrated. Moreover, the IPI could be adapted and evaluated for further wound types, due to the application on different wound types already accomplished during development.

Overall, based on theoretical medical hypotheses, the IPI could be presented and implemented within an automatic algorithm. Hereby, the aim was to detect and predict potential wound infections to improve the wound healing process and the diagnostics of chronic wounds.

## 5. Conclusions

In this work, with the help of clinical expert knowledge and a test data set, an evaluation scheme for wounds was first developed which—in contrast to already established methods—is based on thermal imaging. The result is an “Infection Probability Index” (IPI). In a second step, the relatively laborious manual steps were successfully automated using image processing methods. Finally, it was proven that the developed IPI correlates with the clinically established inflammation marker “CRP”. However, significant IPI differences were found between “infected” vs. “non-infected” wounds only in the subgroup of slightly to moderately elevated CRPs, but not in wounds with strongly elevated CRPs.

## Figures and Tables

**Table 1:** Infection Probability Index (IPI).

Parameter	Name: Description	Weight
Cold spots	None: No cold spot within the wound base or nearby the wound	0
	Particular: 3 or less cold spots within wound base or nearby	2
	Distinct: More than 3 cold spots within wound base or nearby	4
Temperature difference	Difference between wound base and intact skin between $1{\;}^{\circ }C$ and $2{\;}^{\circ }C$	0
	Difference between wound base and intact skin < $1{\;}^{\circ }C$	1
	Difference between wound base and intact skin > $2{\;}^{\circ }C$	2
Temperature distribution	Homogeneous: >50% of temperature fluctuations below $1.2{\;}^{\circ }C$	0
	Inhomogeneous: >50% of temperature fluctuations above $1.2{\;}^{\circ }C$	1
	Concentrated: 3 or less regions of mean temperature above $1.5{\;}^{\circ }C$	1
Wound Margin	Discontinuing: Thermal margin crosses the anatomical margin	1
IPI (Infection probability index):		∑

**Table 2:** Overview of the included patients and wounds (without test data).

	Wounds			Images				
**Patients**	**Laparotomy**	**Pressure Injury**	**Stoma**	**Laparotomy**	**Pressure Injury**	**Stoma**	**Total**	**CRP**
ID1	1	-	1	17	-	10	27	70.98
ID2	-	-	1	-	-	3	3	51.58
ID3	1	-	1	4	-	2	6	55.88
ID4	-	-	1	-	-	3	3	17.13
ID5	1	1	1	8	3	4	15	147.87
ID6	-	1	-	-	2	-	2	11.1
ID7	-	-	1	-	-	4	4	100.95
total	3	2	6	29	5	26	60	

## Data Availability

Data sharing is not applicable to this article.

## Abbreviations

The following abbreviations are used in this manuscript:

CRP	C-reactive protein
IPI	Infection Probability Index
NFC	Near field communication
SSI	Surgical site infection

## References

[1] Sun H., Pulakat L., Anderson D.W. (2020). Challenges and New Therapeutic Approaches in the Management of Chronic Wounds. Curr. Drug Targets.

[2] Olsson M., Järbrink K., Divakar U., Bajpai R., Upton Z., Schmidtchen A., Car J. (2019). The humanistic and economic burden of chronic wounds: A systematic review: The burden of chronic wounds. Wound Repair Regen..

[3] Heyer K., Herberger K., Protz K., Glaeske G., Augustin M. (2016). Epidemiology of chronic wounds in Germany: Analysis of statutory health insurance data: Epidemiology of chronic wounds in Germany. Wound Repair Regen..

[4] Raeder K., Jachan D.E., Müller-Werdan U., Lahmann N.A. (2020). Prevalence and risk factors of chronic wounds in nursing homes in Germany: A Cross-Sectional Study. Int. Wound J..

[5] Nussbaum S.R., Carter M.J., Fife C.E., DaVanzo J., Haught R., Nusgart M., Cartwright D. (2018). An Economic Evaluation of the Impact, Cost, and Medicare Policy Implications of Chronic Nonhealing Wounds. Value Health.

[6] Stülpnagel C.C., Silva N., Augustin M., Montfrans C., Fife C., Fagerdahl A., Gamus A., Klein T.M., Blome C., Sommer R. (2021). Assessing the quality of life of people with chronic wounds by using the cross-culturally valid and revised Wound-QoL questionnaire. Wound Repair Regen..

[7] Gomes A., Teixeira C., Ferraz R., Prudêncio C., Gomes P. (2017). Wound-Healing Peptides for Treatment of Chronic Diabetic Foot Ulcers and Other Infected Skin Injuries. Molecules.

[8] Zhao R., Liang H., Clarke E., Jackson C., Xue M. (2016). Inflammation in Chronic Wounds. Int. J. Mol. Sci..

[9] Sommer K., Sander A.L., Albig M., Weber R., Henrich D., Frank J., Marzi I., Jakob H. (2013). Delayed Wound Repair in Sepsis Is Associated with Reduced Local Pro-Inflammatory Cytokine Expression. PLoS ONE.

[10] Sen C.K., Gordillo G.M., Roy S., Kirsner R., Lambert L., Hunt T.K., Gottrup F., Gurtner G.C., Longaker M.T. (2009). Human skin wounds: A major and snowballing threat to public health and the economy. Wound Repair Regen..

[11] Punchard N.A., Whelan C.J., Adcock I. (2004). The Journal of Inflammation. J. Inflamm..

[12] Kramer A., Dissemond J., Kim S., Willy C., Mayer D., Papke R., Tuchmann F., Assadian O. (2018). Consensus on Wound Antisepsis: Update 2018. Skin Pharmacol. Physiol..

[13] Rodrigues M., Kosaric N., Bonham C.A., Gurtner G.C. (2019). Wound Healing: A Cellular Perspective. Physiol. Rev..

[14] Pang M., Zhu M., Lei X., Xu P., Cheng B. (2019). Microbiome Imbalances: An Overlooked Potential Mechanism in Chronic Nonhealing Wounds. Int. J. Low. Extrem. Wounds.

[15] Brown M.S., Ashley B., Koh A. (2018). Wearable Technology for Chronic Wound Monitoring: Current Dressings, Advancements, and Future Prospects. Front. Bioeng. Biotechnol..

[16] Fierheller M., Sibbald R.G. (2010). A clinical investigation into the relationship between increased periwound skin temperature and local wound infection in patients with chronic leg ulcers. Adv. Ski. Wound Care.

[17] Liu C., van der Heijden F., Klein M.E., van Baal J.G., Bus S.A., van Netten J.J. (2013). Infrared dermal thermography on diabetic feet soles to predict ulcerations: A case study. Advanced Biomedical and Clinical Diagnostic Systems XI.

[18] Bryant R.A., Nix D.P. (2012). Acute & Chronic Wounds: Current Management Concepts.

[19] Florczak B., Scheurich A., Croghan J., Sheridan P., Kurtz D., McGill W., McClain B. (2012). An observational study to assess an electronic point-of-care wound documentation and reporting system regarding user satisfaction and potential for improved care. Ostomy/Wound Manag..

[20] Dong W., Nie L.J., Wu M.J., Xie T., Liu Y.K., Tang J.J., Dong J.Y., Qing C., Lu S.L. (2019). WoundCareLog APP—A new application to record wound diagnosis and healing. Chin. J. Traumatol. = Zhonghua Chuang Shang Za Zhi.

[21] Jordan S., McSwiggan J., Parker J., Halas G.A., Friesen M. (2018). An mHealth App for Decision-Making Support in Wound Dressing Selection (WounDS): Protocol for a User-Centered Feasibility Study. JMIR Res. Protoc..

[22] Jacob C., Sanchez-Vazquez A., Ivory C. (2020). Factors Impacting Clinicians’ Adoption of a Clinical Photo Documentation App and its Implications for Clinical Workflows and Quality of Care: Qualitative Case Study (Preprint). JMIR mHealth uHealth.

[23] Do Khac A., Jourdan C., Fazilleau S., Palayer C., Laffont I., Dupeyron A., Verdun S., Gelis A. (2021). mHealth App for Pressure Ulcer Wound Assessment in Patients with Spinal Cord Injury: Clinical Validation Study. JMIR mHealth uHealth.

[24] Pires I.M., Garcia N. Wound Area Assessment using Mobile Application. Proceedings of the International Conference on Biomedical Electronics and Devices.

[25] Mamone V., Fonzo M.D., Esposito N., Ferrari M., Ferrari V. (2020). Monitoring Wound Healing with Contactless Measurements and Augmented Reality. IEEE J. Transl. Eng. Health Med..

[26] Hani A.F.M., Eltegani N.M., Hussein S.H., Jamil A., Gill P., Hutchison D., Kanade T., Kittler J., Kleinberg J.M., Mattern F., Mitchell J.C., Naor M., Nierstrasz O., Pandu Rangan C., Steffen B. (2009). Assessment of Ulcer Wounds Size Using 3D Skin Surface Imaging. Visual Informatics: Bridging Research and Practice.

[27] Körber A., Rietkötter J., Grabbe S., Dissemond J. (2006). Three-dimensional documentation of wound healing: First results of a new objective method for measurement. JDDG.

[28] Yee A., Harmon J., Yi S. (2016). Quantitative Monitoring Wound Healing Status Through Three-dimensional Imaging on Mobile Platforms. J. Am. Coll. Clin. Wound Spec..

[29] Wang S., Zhang Q., Huang W., Tian H., Hu J., Cheng Y., Peng Y. (2018). A New Smart Mobile System for Chronic Wound Care Management. IEEE Access.

[30] Wang S.C., Anderson J.A.E., Evans R., Woo K., Beland B., Sasseville D., Moreau L. (2017). Point-of-care wound visioning technology: Reproducibility and accuracy of a wound measurement app. PLoS ONE.

[31] Derakhshandeh H., Kashaf S.S., Aghabaglou F., Ghanavati I.O., Tamayol A. (2018). Smart Bandages: The Future of Wound Care. Trends Biotechnol..

[32] Cho H.W., Yoon J.H., Yoo S.S., Choi B.G., Yoo H.J. A Batteryless Chronic Wound Monitoring System with NFC. Proceedings of the IEEE Eurasia Conference on Biomedical Engineering, Healthcare and Sustainability (ECBIOS).

[33] Sattar H., Bajwa I.S., Amin R.U., Sarwar N., Jamil N., Malik M.G.A., Mahmood A., Shafi U. (2019). An IoT-Based Intelligent Wound Monitoring System. IEEE Access.

[34] Hsu J.T., Chen Y.W., Ho T.W., Tai H.C., Wu J.M., Sun H.Y., Hung C.S., Zeng Y.C., Kuo S.Y., Lai F. (2019). Chronic wound assessment and infection detection method. BMC Med. Inform. Decis. Mak..

[35] Barone S., Paoli A., Razionale A.V. (2011). Assessment of Chronic Wounds by Three-Dimensional Optical Imaging Based on Integrating Geometrical, Chromatic, and Thermal Data. Proc. Inst. Mech. Eng. Part H J. Eng. Med..

[36] Dini V., Salvo P., Janowska A., Di Francesco F., Barbini A., Romanelli M. (2015). Correlation Between Wound Temperature Obtained with an Infrared Camera and Clinical Wound Bed Score in Venous Leg Ulcers. Wounds Compend. Clin. Res. Pract..

[37] Chanmugam A., Langemo D., Thomason K., Haan J., Altenburger E.A., Tippett A., Henderson L., Zortman T.A. (2017). Relative Temperature Maximum in Wound Infection and Inflammation as Compared with a Control Subject Using Long-Wave Infrared Thermography. Adv. Ski. Wound Care.

[38] Kraft C.N., Krüger T., Westhoff J., Lüring C., Weber O., Wirtz D.C., Pennekamp P.H. (2011). CRP and leukocyte-count after lumbar spine surgery: Fusion vs. nucleotomy. Acta Orthop..

[39] Sproston N.R., Ashworth J.J. (2018). Role of C-Reactive Protein at Sites of Inflammation and Infection. Front. Immunol..

[40] Pepys M.B., Hirschfield G.M. (2003). C-reactive protein: A critical update. J. Clin. Investig..

[41] László I., Trásy D., Molnár Z., Fazakas J. (2015). Sepsis: From Pathophysiology to Individualized Patient Care. J. Immunol. Res..

[42] Li S., Mohamedi A.H., Senkowsky J., Nair A., Tang L. (2020). Imaging in Chronic Wound Diagnostics. Adv. Wound Care.

[43] Saghaleini S.H., Dehghan K., Shadvar K., Sanaie S., Mahmoodpoor A., Ostadi Z. (2018). Pressure Ulcer and Nutrition. Indian J. Crit. Care Med. Peer-Rev. Off. Publ. Indian Soc. Crit. Care Med..

[44] Boyko T.V., Longaker M.T., Yang G.P. (2018). Review of the Current Management of Pressure Ulcers. Adv. Wound Care.

[45] Zaidi S.R.H., Sharma S. (2021). Decubitus Ulcer. StatPearls.

[46] Amir Y., Lohrmann C., Halfens R.J., Schols J.M. (2017). Pressure ulcers in four Indonesian hospitals: Prevalence, patient characteristics, ulcer characteristics, prevention and treatment: Pressure ulcers in four Indonesian hospitals. Int. Wound J..

[47] (2019). Prevention and Treatment of Pressure Ulcers/Injuries: Quick Reference Guide.

[48] Mervis J.S., Phillips T.J. (2019). Pressure ulcers: Prevention and management. J. Am. Acad. Dermatol..

[49] Tran D.T.A., Klotz R., Harnoss J.C., Heger P., Ritter A.S., Doerr-Harim C., Knebel P., Schneider M., Büchler M.W., Diener M.K. (2021). Standard of Care and Outcomes of Primary Laparotomy Versus Laparotomy in Patients with Prior Open Abdominal Surgery (ReLap Study; DRKS00013001). J. Gastrointest. Surg..

[50] Ahmed A., Azim A. (2020). Emergency Laparotomies: Causes, Pathophysiology, and Outcomes. Indian J. Crit. Care Med..

[51] Abebe K., Bekele M., Tsehaye A., Lemmu B., Abebe E. (2019). Laparotomy for Abdominal Injury Indication & Outcome of patients at a Teaching Hospital in Addis Ababa, Ethiopia. Ethiop. J. Health Sci..

[52] Sahebally S.M., McKevitt K., Stephens I., Fitzpatrick F., Deasy J., Burke J.P., McNamara D. (2018). Negative Pressure Wound Therapy for Closed Laparotomy Incisions in General and Colorectal Surgery: A Systematic Review and Meta-analysis. JAMA Surg..

[53] Vilz T.O., v. Websky M., Kalff J.C., Stoffels B. (2020). Intestinale Stomata. Der Chir..

[54] Tsujinaka S., Tan K.Y., Miyakura Y., Fukano R., Oshima M., Konishi F., Rikiyama T. (2020). Current Management of Intestinal Stomas and Their Complications. J. Anus Rectum Colon.

[55] Grubišić I., Gjenero L., Lipić T., Sović I., Skala T. Active 3D scanning based 3D thermography system and medical applications. Proceedings of the 34th International Convention MIPRO.

[56] Beauvisage A., Aouf N. Low cost and low power multispectral thermal-visible calibration. Proceedings of the IEEE SENSORS.

[57] Divya P., Anusudha K. Segmentation of Defected Regions in Leaves using K- Means and OTSU’s Method. Proceedings of the 4th International Conference on Electrical Energy Systems (ICEES).

[58] Johnson T.R., Gómez B.I., McIntyre M.K., Dubick M.A., Christy R.J., Nicholson S.E., Burmeister D.M. (2018). The Cutaneous Microbiome and Wounds: New Molecular Targets to Promote Wound Healing. Int. J. Mol. Sci..

[59] Chen H., Ding H., He X., Zhuang H. Color image segmentation based on seeded region growing with Canny edge detection. Proceedings of the 12th International Conference on Signal Processing (ICSP).

[60] Narkhede P.R., Gokhale A.V. Color image segmentation using edge detection and seeded region growing approach for CIELab and HSV color spaces. Proceedings of the International Conference on Industrial Instrumentation and Control (ICIC).

[61] Yu Y.-W., Wang J.-H. Image segmentation based on region growing and edge detection. Proceedings of the 1999 IEEE International Conference on Systems, Man, and Cybernetics (Cat. No.99CH37028).

[62] Susan S., Verma O.P., Swarup J. Object Segmentation by an Automatic Edge Constrained Region Growing Technique. Proceedings of the Fourth International Conference on Computational Intelligence and Communication Networks.

[63] Shimbashi T., Kokubo Y., Shirota N. Region segmentation using edge based circle growing. Proceedings of the International Conference on Image Processing.

[64] Rashid M.H.O., Mamun M.A., Hossain M.A., Uddin M.P. Brain Tumor Detection Using Anisotropic Filtering, SVM Classifier and Morphological Operation from MR Images. Proceedings of the International Conference on Computer, Communication, Chemical, Material and Electronic Engineering (IC4ME2).

[65] Childs C., Wright N., Willmott J., Davies M., Kilner K., Ousey K., Soltani H., Madhuvrata P., Stephenson J. (2019). The surgical wound in infrared: Thermographic profiles and early stage test-accuracy to predict surgical site infection in obese women during the first 30 days after caesarean section. Antimicrob. Resist. Infect. Control.

[66] Schaefer G., Tait R., Howell K., Hopgood A., Woo P., Harper J. (2008). Automated Overlay of Infrared and Visual Medical Images. User Centered Design for Medical Visualization.

[67] Soerensen D.D., Pedersen L.J. (2015). Infrared skin temperature measurements for monitoring health in pigs: A review. Acta Vet. Scand..

